# Correlation between the presence of tonsilloliths and the bone defects by periodontitis on imaging analysis: a pilot study

**DOI:** 10.1186/s12903-023-03769-3

**Published:** 2024-01-03

**Authors:** Masafumi Oda, Shinji Yoshii, Nao Wakasugi-Sato, Shinobu Matsumoto-Takeda, Ikuko Nishida, Shun Nishimura, Susumu Nishina, Manabu Habu, Daigo Yoshiga, Masaaki Sasaguri, Yasuhiro Morimoto

**Affiliations:** 1 Division of Oral and Maxillofacial Radiology, Kyushu Dental University, 2-6-1 Manazuru, Kitakyushu, Kokurakita-ku 803-8580 Japan; 2 Division of Promoting Learning Design Education, Kyushu Dental University, Kitakyushu, Japan; 3 Division of Developmental Stomatognathic Function Science, Kyushu Dental University, Kitakyushu, Japan; 4 Division of Maxillofacial Surgery, Kyushu Dental University, Kitakyushu, Japan; 5 Division of Oral Medicine, Kyushu Dental University, Kitakyushu, Japan

**Keywords:** Tonsilloliths, CT, Periodontitis, Panoramic radiographs

## Abstract

**Background:**

Very recently, a significant relationship between tonsilloliths and dental plaque-related pathologies was reported using digital panoramic radiographs. Their dynamics over time suggest that tonsilloliths may be in a permanently active phase that functions to remove foreign matter. The aim of the study was to evaluate the relationship between the occurrence of tonsilloliths and the extent of periodontitis.

**Methods:**

A total of 608 patients who underwent both CT and panoramic radiographs were included in the study. Both of two imaging were retrospectively and independently assessed with respect to the presence of tonsilloliths detected on CT and panoramic radiographs, and bone defects caused by periodontitis detected on panoramic radiographs. The type of retrospective study is case-control. Then, the differences between age groups were evaluated with respect to the degree of bone resorption and its correlation with the presence of tonsilloliths. The relationships between categorical variables were assessed using Pearson’s correlation coefficient or Spearman’s correlation coefficient.

**Results:**

There was a significant relationship between tonsilloliths on CT and the extent of the bone defect on panoramic radiographs (Spearman’s correlation coefficient, *r* = 0.648, *p* = 0.043). In addition, there was a significant difference in the extent of the bone defect caused by periodontitis between subjects with and without tonsilloliths in the 60 to 69-year-old group (Mann-Whitney U test, *p* = 0.025), 70 to 79-year-old group (Mann-Whitney U test, *p* = 0.002), and 80 to 89-year-old group (Mann-Whitney U test, *p* = 0.022), but not in other age groups (Mann-Whitney U test: under 9-year-old group, *p* = 1.000; 10 to 19-year-old group, *p* = 1.000; 20 to 29-year-old group, *p* = 0.854; 30 to 39-year-old group, *p* = 0.191, 40 to 49-year-old group, *p* = 0.749; 50 to 59-year-old group, *p* = 0.627; ≥90-year-old group, *p* = 1.000).

**Conclusions:**

The presence of tonsilloliths was related to the extent of periodontitis because the structures were responding dynamically.

## Background

Tonsilloliths are oropharyngeal concretions that form within the palatine tonsillar crypt in reaction to a foreign nidus such as bacteria or organic debris [1]. It has been suggested that tonsilloliths may be correlated with halitosis and tonsillar abscess [2–9]. Tonsilloliths have very high detection rates on computed tomography (CT), such as nearly 50% in some reports [2, 10–14]. A recent longitudinal study of changes in tonsilloliths found dynamic fluctuation of the calcification levels of all tonsilloliths with time [10]. It was hypothesized that their dynamics over time suggest that tonsilloliths may be in a permanently active phase that functions to remove foreign matter [10].

Very recently, a significant relationship between tonsilloliths and dental plaque-related pathologies was reported buy Gurbiuz et al. using digital panoramic radiographs [15]. In addition, some of the bacteria as a foreign nidus in tonsilloliths are periodontal disease-related bacteria, such as *Actinomyces, Eubacterium, Fusobacterium, Megasphaera, Porphyromonas, Prevotella, Selenomonas,* and *Tannerella* spp. [16–18]. These results seem reasonable because tonsilloliths may be involved in continuously dental plaque-related pathologies such as periodontal disease and active alteration of calcifications, which are correlated to the surrounding environment [10, 15]. However, the report by Gurbuz et al. was relatively limited because the presence or absence of tonsilloliths was judged by panoramic radiographs in that study [15]. The detection rate of tonsilloliths by panoramic radiographs was only about 13%, not approaching the nearly 50% seen on CT [2, 10, 15].

Therefore, it was thought that it necessary to evaluate patients’ tonsilloliths using CT in order to investigate the relationship between tonsilloliths and periodontal disease more precisely. Then it was retrospectively hypothesized that analyzing the relationship between the presence or absence of tonsilloliths on CT and bone loss due to periodontal disease on panoramic radiographs would be effective in clarifying the relationship between the two.

## Methods

A total of 608 patients who underwent both pre-treatment CT including the palatine tonsils and panoramic radiographs were included in the retrospective study. The type of retrospective study is case-control. No CT or panoramic radiographs were performed for this study. The examination was performed at the Division of Oral and Maxillofacial Radiology of Kyushu Dental University between 2020 and 2022. Patients who underwent CT including the palatine tonsils and panoramic radiographs had maxillary and mandibular disease such as inflammation, cysts, tumors, and etc. of the soft tissues of the maxilla, mandible, and head and neck, and required both examinations clinically. Patients who had no remaining teeth and patients without pre-treatment CT and panoramic radiographs, low quality panoramic radiograph and low quality CT image due to metal artifact to calculate the presence of tonsillolith were excluded. In addition, CBCT and panoramic radiographs together were not used is that CBCT cannot adequately evaluate the tonsilloliths. Patients involved in this study were 237 male and 371 female patients. Patients ranged in age from 5 to 94 years, with a mean of 50.3 years. The present study was approved by the institutional review board (IRB) of Kyushu Dental University (No. 20–16). This study is retrospective, and the Kyushu Dental University the IRB has indicated that informed consent from patients for this study is not required.

Panoramic radiographs were acquired using a panoramic AUTO-1000 EX system (Asahi Roentgen Ind. Co., Ltd., Kyoto, Japan). Images were taken in the incisive occlusion position with the head held by head supports with the FH plane parallel to the ground. CT was performed using an Activion 16 (Toshiba, Tokyo, Japan) scanner. Contiguous axial images with slice thickness of 3 mm were obtained using standard algorithms and viewed with soft-tissue windows.

**Fig. 1:**
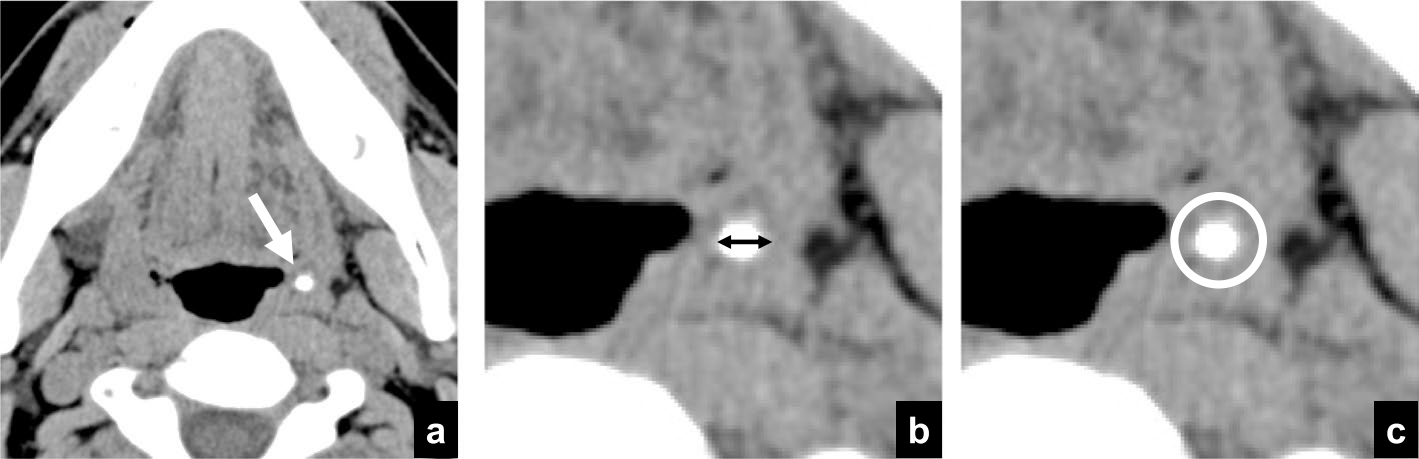
Methods for measurement of sizes and CT values of tonsilloliths on CT images. **a** The largest calculus (soft-tissue window setting, arrow) in the palatine tonsil is included in the analysis. **b** Size is defined as the long axis measurement (arrow) on the soft-tissue window setting. **c** The calcification level is defined as the maximum CT value on the soft-tissue window setting, and the region of interest includes the whole calculus (circle)

The presence of tonsilloliths was evaluated independently on both panoramic radiographs and CT. Using the differential diagnosis of tonsilloliths on panoramic radiographs reported by Ram et al., a radiopaque nodular mass or masses piled up on the mandibular ramus and soft palate were defined as tonsilloliths [2, 19]. Tonsillolith pathology was diagnosed based on the system of Oda et al., as follows [2] **(**Fig. 1**)**. Tonsilloliths were defined as structures of CT density > 100 Hounsfield units (HU). The numbers and long axes of the tonsilloliths were calculated on CT images. The calcification levels of the tonsilloliths were calculated as CT values. If the subject had multiple tonsilloliths, the size and calcification levels of the largest calculus were analyzed. Analysis of tonsillolith on 608 pairs of panoramic radiographs and CT images were assessed by a single oral and maxillofacial radiologist with 15 years of experience (M. O.). To reduce sources of bias, the investigator for tonsilloliths was a single experienced oral and maxillofacial radiologist familiar with the study.

**Fig. 2:**
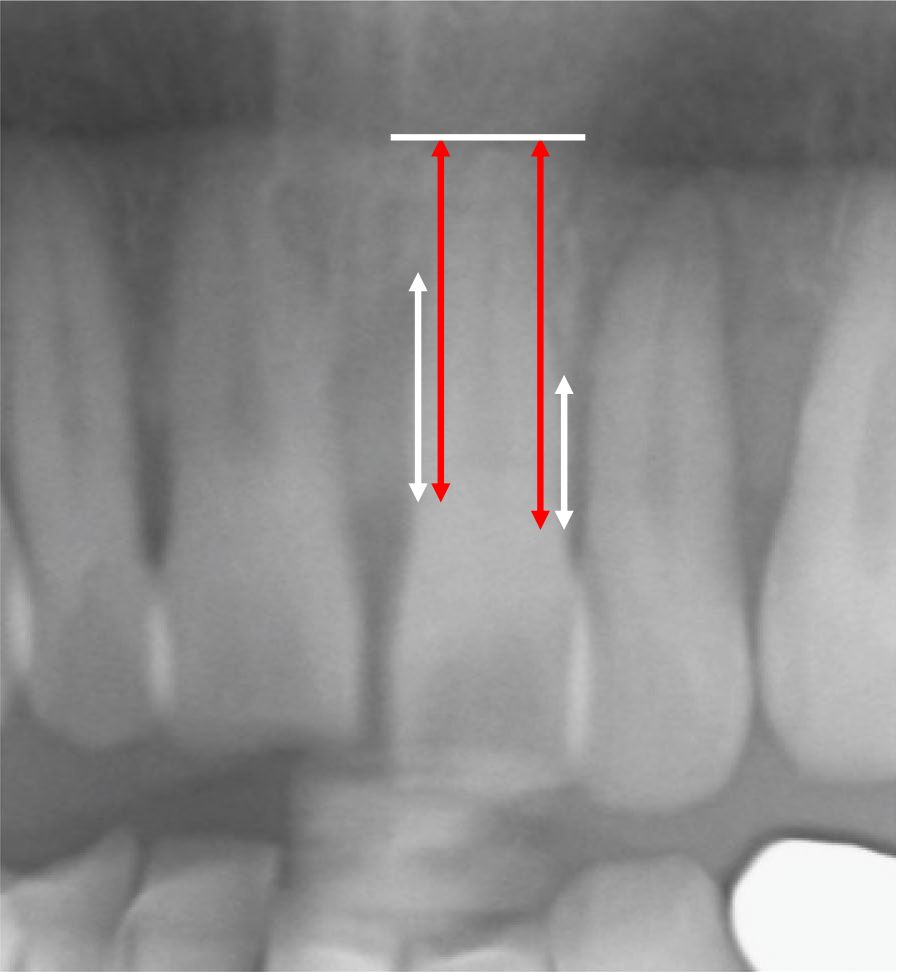
Methods for assessing periodontal bone defects caused by periodontitis on panoramic radiographs. A cropped panoramic tomograph is shown. The alveolar bone defect caused by periodontitis is assessed on a panoramic radiograph by measuring the distance between the cement-enamel junction and the alveolar crest (arrows) and between the cement-enamel junction and the root apex (red arrows) at mesial and distal sites on each tooth. The alveolar bone defect caused by periodontitis of each subject is calculated as the average of the cement-enamel junction-alveolar crest/cement-enamel junction-apex of all teeth

Alveolar bone defects caused by periodontitis were assessed using panoramic radiographs, which were commonly used to evaluate bone loss due to periodontal disease, that included all teeth. Implants, supernumerary teeth, and third molars were excluded from the number of teeth. Residual roots without a cap for an overdenture were also excluded. Teeth with caries or periapical lesions were not excluded. Bone loss was considered when the alveolar crest was found to be 2 mm or more from the cemento-enamel junction according to Gurbuz et al. [15]. Alveolar bone defects caused by periodontitis were assessed on panoramic radiographs by measuring the distance between the cement-enamel junction and the alveolar crest and between the cement-enamel junction and the root apex at two sites (mesial and distal) on each tooth **(**Fig. 2**)** [20]. The apex was defined as the most apically located point of the root. The alveolar bone defect caused by periodontitis of each subject was calculated as the average of the cement-enamel junction-alveolar crest/cement-enamel junction-apex of all teeth [21]. The points of cement-enamel junction, alveolar crest and apex of teeth on panoramic radiograph were assessed by an oral and maxillofacial radiologist with 15 years of experience (M. O.) and a conservative dentist with 14 years of experience (S. Y.). The measurement point was determined by agreement between the two investigators. To reduce sources of bias, those assessing for alveolar bone defects on panoramic radiographs were performed by experts of radiologists and conservative dentist. In addition, CT and panoramic radiographs were evaluated independently and randomly.

The essential null hypothesis of this study is that the presence of tonsilloliths on CT is not related to alveolar bone loss on panoramic radiographs. On the other hand, the alternative hypothesis is that there is a relationship between the two. Sample size was calculated with using G*Power version 3.1.9.6 (Franz Faul, University of Kiel, Germany). Input parameters and sample sizes were as follows: tails, two; effect size, 0.3; alpha error, 0.05; power (1-Beta error): 0.95; allocation ratio N2/N1, 1.2. And 586 was calculated as the sample size. All statistical analyses were performed using IBM SPSS Statistics version 26 statistical software (SPSS, Chicago, IL, USA). Categorical variables were compared using Fisher’s exact test or the Mann-Whitney U test. The relationships between categorical variables were assessed using Pearson’s correlation coefficient or Spearman’s correlation coefficient. Results were considered significant at *p* < 0.05. The sample size was consulted with a statistician to ensure that it was sufficient for this study.

## Results

### Detection of tonsilloliths on CT and panoramic radiographs

Of the 608 individuals, 278 (45.7%) were considered to have tonsilloliths on CT scans, with only 64 (10.5%) on panoramic radiographs. The 278 patients with tonsilloliths on CT consisted of 107 males and 171 females, with no difference in the tonsillolith detection rate by sex on CT (Fisher’s exact test, *p* = 0.867) and on panoramic radiographs (Fisher’s exact test, *p* = 0.685). A significant correlation was found between age and the tonsillolith detection rate on CT (Spearman’s correlation coefficient, *r* = 0.894, *p* < 0.001) (Table 1). As the subjects aged, the detection rate increased gradually. In particular, a significant difference in the tonsillolith detection rate was found between the under and over 40-year-old age groups (Fisher’s exact test, *p* < 0.001).

**Table 1:** Age distribution for the detection of tonsilloliths on CT and the extent of the bone defect caused by periodontitis calculated on panoramic tomography

Age (y)	Present		Absent		Mann-Whitney U test (*p* value)	Detection rate (%)
	Number of patients	Average bone loss (%)	Number of patients	Average bone loss (%)		
≤9	4	0	4	0	1.000	50.0
10–19	7	0	32	0	1.000	17.9
20–29	32	1.1	63	1.8	0.854	33.7
30–39	28	5.2	55	3.4	0.191	33.7
40–49	31	10.1	31	9	0.749	50.0
50–59	44	17.4	35	19	0.627	55.7
60–69	42	31.7	41	21.9	0.025*	50.6
70–79	58	33.2	46	20.1	0.002*	55.8
80–89	30	32.4	22	23.8	0.022*	57.7
≥90	2	44.7	1	7.5	1.000	66.7
Total	278	20.1	330	11.2	< 0.001*	45.7

*p < 0.05

Overall, 105 patients had one calculus, 48 had two, 39 had three, 23 had four, 18 had five, and so on (Table 2), up to a maximum of 36. With respect to the sizes of the tonsilloliths, 76 were less than 2 mm, 188 were greater than 2 mm and less than 5 mm, 14 were greater than 5 mm, and none was greater than 8 mm (Table 3). The calcification levels of tonsilloliths on CT images were greater than 100 HU and less than 300 HU in 115, greater than 300 HU and less than 500 HU in 77, greater than 500 HU and less than 1000 HU in 66, and greater than 1000 HU in 20 (Table 4).

**Table 2:** Distribution of the number of tonsilloliths on CT and the extent of the bone defect caused by periodontitis calculated on panoramic tomography

Number of tonsilloliths	Number of patients	Average bone loss (%)
0	330	11.2
1	105	17.1
2	48	26.9
3	39	24.4
4	23	15.3
5	18	16.3
6	8	16.4
7	8	33.8
8	8	11.9
9	4	19.5
10	4	12.6
11	5	6.3
≥12	8	30.1
		(*n* = 608)

**Table 3:** Distribution of the sizes of tonsilloliths on CT and the extent of the bone defect caused by periodontitis calculated on panoramic tomography

Size of tonsilloliths (mm)	Number of patients	Average bone loss (%)
< 1	8	14.7
1 - < 2	68	20.4
2 - < 3	64	20.2
3 - < 4	78	17.4
4 - < 5	46	24.3
5 - < 6	9	17.6
6 - < 7	3	38.3
7 - < 8	2	15.7
		(*n* = 278)

**Table 4:** Distribution of calcification levels of tonsilloliths on CT and the degree of the bone defect caused by periodontitis calculated on panoramic tomography

Calcification level (HU)	Number of patients	Average bone loss (%)
< 200	62	13.8
200 - < 300	53	28.5
300 - < 400	38	20.8
400 - < 500	39	15.3
500 - < 600	22	21
600 - < 700	15	20.4
700 - < 800	12	18.5
800 - < 900	11	26.2
900 - < 1000	6	14.8
1000 - < 1100	5	9.1
1100 - < 1200	5	45.6
≥1200	10	18.9
		(*n* = 278)

### Relationship between the detection of tonsilloliths on CT and the extent of the bone defect caused by periodontitis on panoramic radiographs

**Fig. 3:**
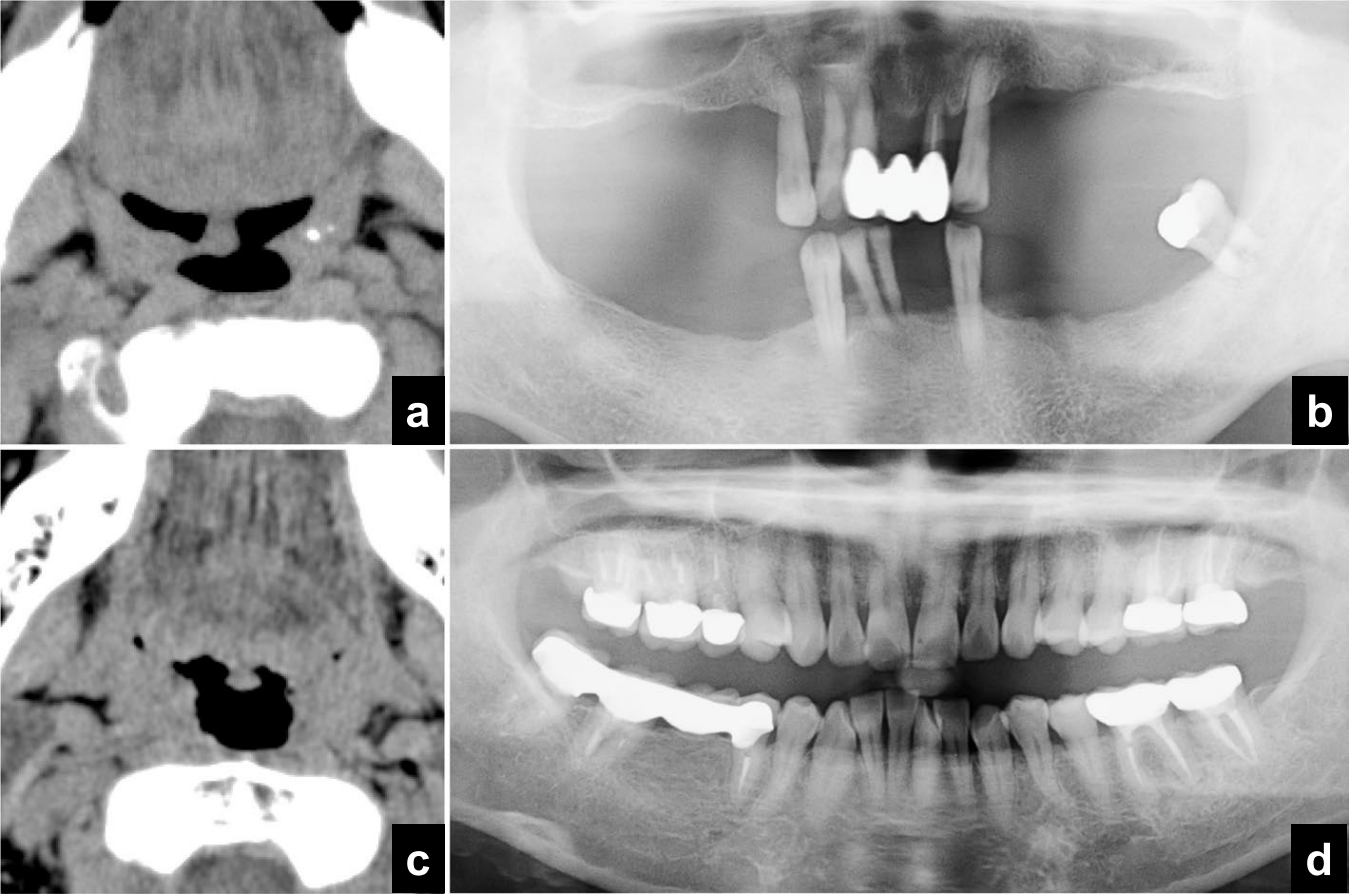
Relationship between the detection of tonsilloliths on CT and the extent of the bone defect caused by periodontitis on panoramic radiographs in the 60s age group. **a** Axial CT images at the palatine tonsillar level showing a tonsillolith in a 63-year-old male patient. **b** Panoramic radiographs showing a severe bone defect caused by periodontitis in the same patient of Fig. 3a. **c** Axial CT images at the palatine tonsillar level showing no tonsillolith in a 63-year-old female patient. **d** Panoramic radiographs showing almost no bone defect caused by periodontitis in the same patient of Fig. 3c

**Fig. 4:**
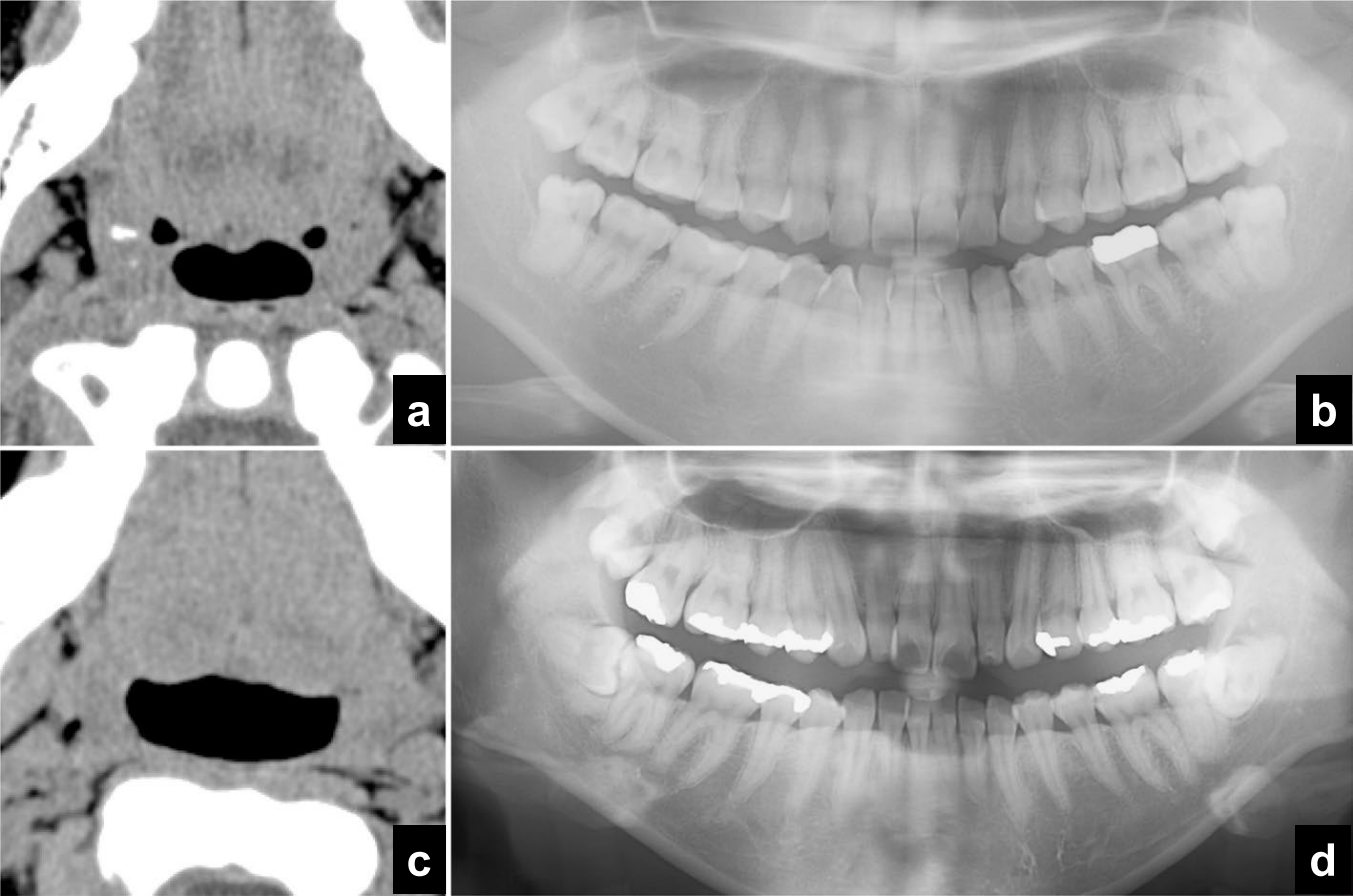
Relationship between the detection of tonsilloliths on CT and the extent of the bone defect caused by periodontitis on panoramic radiographs in the 30s age group. **a** Axial CT images at the palatine tonsillar level showing a tonsillolith in a 32-year-old female patient. **b** Panoramic radiographs showing almost no bone defect by periodontitis in the same patient of Fig. 4a. **c** Axial CT images at the palatine tonsillar level showing no tonsillolith in a 32-year-old female patient. **d** Panoramic radiographs showing almost no bone defect caused by periodontitis in the same patient of Fig. 4c

A significant difference in the extent of the bone defect caused by periodontitis was seen between subjects with and without tonsilloliths (Mann-Whitney U test, *p* < 0.001) (Table 1). A strong correlation was found between the tonsillolith detection rate on CT and the extent of the bone defect caused by periodontitis (Spearman’s correlation coefficient, *r* = 0.648, *p* = 0.043) (Table 5) (Fig. 3). As the extent of the bone defect caused by periodontitis increased, the detection rate increased gradually. There was a significant difference in the extent of the bone defect caused by periodontitis between subjects with and without tonsilloliths in the 60 to 69-year-old group (Mann-Whitney U test, *p* = 0.025) (Fig. 3), 70 to 79-year-old group (Mann-Whitney U test, *p* = 0.002), and 80 to 89-year-old group (Mann-Whitney U test, *p* = 0.022), but not in other age groups (Mann-Whitney U test: under 9-year-old group, *p* = 1.000; 10 to 19-year-old group, *p* = 1.000; 20s, *p* = 0.854; 30 to 39-year-old group, *p* = 0.191 (Fig. 4), 40 to 49-year-old group, *p* = 0.749; 50 to 59-year-old group, *p* = 0.627; ≥90-year-old group, *p* = 1.000) (Table 1). The number of tonsilloliths on CT was weakly related to the bone defect caused by periodontitis (Spearman’s correlation coefficient, *r* = 0.235, *p* < 0.001) (Table 2). No significant correlations were found between the extent of the bone defect caused by periodontitis on panoramic radiographs and the size of tonsilloliths (Pearson’s correlation coefficient, *r* = 0.45, *p* = 0.458) (Table 3) or HU number (Pearson’s correlation coefficient, *r* = 0.020, *p* = 0.746) (Table 4).

**Table 5:** Distribution of the extent of the bone defect caused by periodontitis calculated on panoramic tomography and the tonsillolith detection rate on CT

Bone loss (%)	Number of patients		Detection rate (%)
	Present	Absent	
0	66	136	32.7
< 10	59	80	42.4
10 - < 20	44	53	45.4
20 - < 30	37	22	62.7
30 - < 40	25	15	62.5
40 - < 50	17	7	70.8
50 - < 60	7	4	63.6
60 - < 70	12	7	63.2
70 - < 80	9	4	69.2
80 - < 90	2	2	50.0
Total	278	330	45.7
			(*n* = 608)

## Discussion

It was retrospectively hypothesized that there is a relationship between the presence of tonsilloliths and the extent of periodontitis. The relationships between the detection rate of tonsilloliths on CT and the extent of the bone defect caused by periodontitis on panoramic radiographs were evaluated by age group. It was found that there was a significant relationship between the detection rate of tonsilloliths on CT and the extent of the bone defect caused by periodontitis on panoramic radiographs. In particular, one of the most interesting results of the present study was that there was a significant correlation between the detection rate of tonsilloliths and the extent of the bone defect caused by periodontitis on panoramic radiographs in the relatively older age group.

In the previous reports, tonsilloliths were shown to form within the palatine tonsillar crypt in reaction to a foreign nidus such as bacteria or organic debris. Some of the bacteria as a foreign nidus are periodontal disease-related bacteria, such as *Actinomyces, Eubacterium, Fusobacterium, Megasphaera, Porphyromonas, Prevotella, Selenomonas,* and *Tannerella* spp. [16, 17]. Oral bacteria in secretions in contact with tonsillar epithelium may adhere to the mucosal surface and may stimulate cytokine production by mucosal epithelium [22]. In a clinical study, it was shown that the prevalence of periodontal disease is increased in patients with peritonsillar abscess [23]. The aforementioned studies emphasized the possibility of a causal relationship for these pathologies. In addition, it was reported that there were dynamic fluctuations of the calcification levels of all tonsilloliths, as well as a tendency for the tonsilloliths to be excreted from the body with increasing tonsillolith size [10]. The active dynamics of tonsilloliths function to remove foreign matter from the body [10]. Together with the present results and previous reports, our hypothesis seems. The status of periodontitis might be related to the occurrence of tonsilloliths in older age groups. It was very recently reported that there was a significant relationship between tonsilloliths and dental plaque-related pathologies on digital panoramic radiographs [15]. As the subjects aged, the detection rate of tonsilloliths and the rate of dental plaque-related pathologies increased gradually [15]. Therefore, the result could not be negated by confounding factors with aging. However, the present study showed that there was a significant correlation between the detection rate of tonsilloliths and the extent of the bone defect on panoramic radiographs in the relatively older age group. The present results mean that there may be a direct relationship between the presence of tonsilloliths and the occurrence of periodontitis, without confounding due to aging.

At the same time, on one side, the results support the relationship between the occurrence of halitosis and the presence of tonsilloliths through periodontitis. Tonsilloliths, also known as smelly balls, have a strong odor that might induce halitosis. So far, there have been some reports of previous studies about the relationship between the occurrence of halitosis and the presence of tonsilloliths [7, 16, 19, 24, 25], but there is no consensus. Since the relationship between the presence of tonsilloliths and the extent of periodontitis was elucidated in the present study, the results support the relationship between the occurrence of halitosis and the presence of tonsilloliths. However, tonsilloliths with a strong odor might not necessarily induce halitosis directly, and the periodontitis coexisting with tonsilloliths might be partially responsible.

The limitations of the recent previous study of the relationship between tonsilloliths and dental plaque-related pathologies might be related to sampling [15]. The detection rate of tonsilloliths was 46.1% on CT scans, but only 7.3% on panoramic radiographs [2]; the main causes for the discrepancy were the calcification levels (CT value < 300 HU) and the sizes (< 5 mm) of the tonsilloliths in the report [2].

Another interesting result was that there was no significant correlation between the detection rate of tonsilloliths and the extent of the bone defect caused by periodontitis on panoramic radiographs in younger and middle-aged groups, but not in the older age group. This result suggests that the extent of the bone defect caused by periodontitis might be partially related to the detection rate of tonsilloliths, but not directly or completely. Dynamic changes of calcification levels of almost all tonsilloliths occur [10]. Therefore, as the rate of various kinds of periodontitis-related bacteria may be increasing, the formation and the growth of tonsilloliths should be affected. An unknown mechanism in addition to hormone concentrations and pH in saliva might affect the dynamic changes in tonsilloliths. Further studies are needed to clarify the reasons for the relationship between the presence of tonsilloliths and the extent of periodontitis.

The major limitations of the present study are that the status of periodontitis was assessed only by the extent of bone defects on panoramic radiographs. The plaque index, the numbers of bleeding on probing, mobility, redness, swelling by inspection, etc. should be examined to determine the status of periodontitis. Because the present study was retrospective, it failed to assess confounding factors that should be considered, even though periodontitis is a multifactorial disease. In particular, the clinical attachment loss and other radiologic examination, which is fundamental to the diagnosis of periodontal disease, were unable to do. However, it is not considered this limitation to be decisive for the validity of the study results. In the future, it is believed that the possible relationship between tonsil stones and periodontitis will be further elucidated by using PCR to examine the relationship between periodontal bacterial strains and those in tonsil stones and by examining the role of nanobacteria in dental calculus and tonsilloliths. In addition, the data were obtained from subjects with diseases mainly involving the oropharyngeal area, rather than the oral and maxillofacial region. The present results should be interpreted as being relevant to relatively healthy, active populations. A further limitation is that only Japanese subjects were examined.

## Conclusions

The aim of the study was to evaluate the relationship between the occurrence of tonsilloliths and the extent of periodontitis. A total of 608 pairs of CT and panoramic radiographs were retrospectively assessed for the presence of tonsilloliths and bone defects caused by periodontitis. There was a significant relationship between tonsilloliths on CT and the extent of the bone defect caused by periodontitis on panoramic radiographs. At the same time, there was a significant correlation between the presence of tonsilloliths and the extent of the bone defect caused by periodontitis on panoramic radiographs in the relatively older age group, but not in the younger or middle-aged population. The presence of tonsilloliths might be partially related to the extent of periodontitis, because the structures might be responding dynamically to a foreign nidus such as bacteria in the body.

## Data Availability

All data generated or analysed during this study are included in this published article.

## Abbreviations

CT: Computed tomography
HU: Hounsfield units
